# A retrospective cohort of smartpilot view–assisted effect-site TCI anaesthesia in primary total hip and knee arthroplasty

**DOI:** 10.1186/s12871-025-03471-7

**Published:** 2025-11-06

**Authors:** Erik Noppa, Alexander Persson, Henrik Öhrström, Per Wretenberg, Eva Lundqvist

**Affiliations:** 1 Faculty of Medicine and Health, Örebro University, Örebro, Sweden; 2 Department of Anaesthesiology and Critical Care, Örebro University Hospital, Lindesberg, Sweden; 3 Department of Hand and Orthopaedic Surgery, Örebro University Hospital, Lindesberg, Sweden

**Keywords:** Anaesthesia outcomes, Enhanced recovery after surgery (ERAS), Perioperative care, Postoperative nausea and vomiting (PONV), Postoperative recovery, Target-controlled infusion, Total hip arthroplasty, Total knee arthroplasty

## Abstract

**Background:**

Primary total hip arthroplasty and total knee arthroplasty are common surgical procedures that require effective perioperative care to ensure early mobilisation, reduced complications, and shortened hospital stay. Effect-site target-controlled infusion (TCI) combined with SmartPilot View (SPV) is a novel approach to total intravenous anaesthesia that may improve anaesthetic precision and reduce postoperative side effects. However, it has not been thoroughly evaluated in joint arthroplasty. The aim of this study was to evaluate the early anaesthetic outcomes in hip and knee arthroplasty patients following the implementation of effect-site TCI and SPV.

**Methods:**

We conducted a retrospective cohort study including 2316 procedures (2210 patients) at Örebro University Hospital, Lindesberg, Sweden, between 2018 and 2023. Two groups were compared: pre-implementation (2018–2019, *n* = 1258) using plasma-targeted TCI without SPV, and post-implementation (2022–2023, *n* = 1058) using effect-site TCI with SPV. Patients from 2020 to 2021 were excluded due to the COVID-19 pandemic and the overlap of methods during implementation. Data were extracted from the Swedish Perioperative Register. The primary outcome was the incidence of postoperative nausea and vomiting (PONV). Secondary outcomes included length of stay in the post-anaesthesia care unit (phase II), postoperative pain, and surgical duration.

**Results:**

PONV incidence decreased significantly from 8.4% to 3.0% after implementation (*p* < 0.001; adjusted OR: 0.36, 95% CI: 0.23–0.55). Median post-anaesthesia care unit stay decreased by 70 min (95% CI: 61–78 min; *p* < 0.001). Postoperative pain scores were slightly lower in the post-implementation group (median numeric rating scale 7 vs. 5; *p* < 0.001). Surgical duration increased marginally (84 vs. 87 min; *p* = 0.003).

**Conclusions:**

Implementation of effect-site TCI and SPV in hip and knee arthroplasty was associated with significantly reduced PONV and shorter post-anaesthesia care unit stay, suggesting improved early postoperative recovery.

**Clinical trial number:**

Not applicable.

## Introduction

Primary total knee arthroplasty (TKA) and total hip arthroplasty (THA) are common and efficient treatments for fractures, complications of congenital diseases, and most commonly osteoarthritis, leading to decreased pain, enhanced quality of life, and reduced mortality [1–3]. The number of arthroplasties has increased since the late 1970 s, creating a high demand for effective and reliable patient care including early mobilisation and often discharge within 24 h [4].

Modern anaesthesia plays a crucial role in reducing complications for these common procedures, enabling early mobilisation and decreasing overall hospital stay. TCI (target-controlled infusion) is a computer-based technique for continuous intravenous drug administration. It uses information about the patient’s age, weight, gender, and height in order to calculate plasma or effect-site concentration on the basis of a pharmacokinetic model [5].

Several pharmacological models have been developed, but studies have found that for propofol the Schnider model is most reliable, and for remifentanil the Minto model is most reliable [6, 7]. TCI can be used in two ways: plasma-targeted TCI (TCIp) and effect-site-targeted TCI (TCIe). TCIp aims to maintain a specific plasma concentration of the drug, whereas TCIe is based on the estimated concentration in the effect site [8]. TCI makes it possible to administer a continuous intravenous infusion of anaesthetic drugs to maintain a specific effect-site concentration without performing complex calculations [9].

SmartPilot View (SPV; Dräger, Lübeck, Germany) is a pharmacologic model-based decision-support system to optimize anaesthetic depth. It can be combined with TCI, and is fully integrated with the Dräger Zeus anaesthesia workstation [10]. SPV is based on both pharmacokinetic and pharmacodynamic simulations to optimize anaesthetic drugs, predict anaesthetic depth, and facilitate early recovery. The system forecasts the effects of hypnotic and analgesic combinations for the next 10–20 min. Studies have indicated that SPV may contribute to faster awakening and extubation after anaesthesia [11]. It reduces the occurrence of hypotension and hypertension during anaesthesia, decreases the average time to awakening, and shortens the time spent in the recovery unit [12, 13]. This is likely due to its ability to anticipate upcoming anaesthetic depth, allowing for reduced doses of anaesthetic agents.

The combination of SPV with TCIe is a new method aimed at improving the precision of drug administration during surgery and minimising side effects. However, we are not aware of any major studies on SPV with TCIe in joint prosthesis surgery or its impact on postoperative nausea and vomiting (PONV) or duration of recovery in a larger clinical setting. Previous studies have included approximately 100 patients.

This study aimed to evaluate the early anaesthetic outcomes in hip and knee arthroplasty patients following the implementation of TCIe and SPV, focusing on PONV, length of stay in the post-anaesthesia care unit (PACU), pain levels, and surgical duration. We hypothesised that implementation of TCIe and SPV would have positive effects on PONV and duration of PACU stay.

## Methods

### Patients and data gathering

This retrospective cohort study included all elective primary THA and TKA at Örebro University Hospital, Lindesberg, Region Örebro County, Sweden from 2018 to 2023. At this hospital, SPV and TCIe were implemented in 2020–2021 to replace TCIp without SPV.

**Fig. 1:**
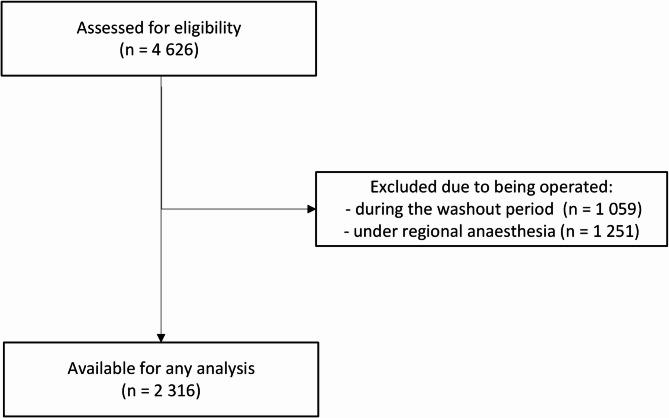
STROBE flowchart

Patients operated during 2020 and 2021 were excluded because of interference from the emergency phase of the COVID-19 pandemic and to achieve a washout period before and after implementation of TCIe and SPV. Only patients operated under general anaesthesia was included because SPV is not applicable in the context of neuraxial anaesthesia and TCIe has limited use. The final cohort for analysis comprised 2316 cases (Fig. 1). The study was approved by the Swedish Ethical Review Authority (ref: 2024–08042-01), and a waiver of informed consent was granted.

All data for this study were extracted from the Swedish Perioperative Register (SPOR), which is a quality register for perioperative care in Sweden with almost complete coverage. The register data has been verified as having high validity [14]. Data used for this study were recorded by staff involved in the care of the patients, either preoperatively by the surgeon or anaesthesiologist, intraoperatively by the operating theatre staff or anaesthesia nurse, or postoperatively by the PACU nurses.

A power analysis was performed assuming a pre-implementation PONV incidence of 9% and a 50% relative reduction post-implementation. To detect this difference with 90% power at a 5% significance level (two-sided), a total of 1,304 patients would be required.

### Perioperative and surgical care

All patients in this study were anaesthetised using propofol and remifentanil with TCI according to clinical routine, which was not changed during this time period except for the implementation of TCIe and SPV. PONV prophylaxis was administered to all patients with 8 mg betamethasone and 4 mg ondansetron intravenously in conjunction with the start of anaesthesia; no changes were made to this during the study period. Airway management was performed with a supraglottic airway device if appropriate, or an endotracheal tube otherwise. Electroencephalogram-based anaesthesia depth monitoring with the bispectral index was used on all patients [15]. Postoperative pain management was unchanged during the study period, and consisted of local infiltration anaesthesia, paracetamol, parecoxib, clonidine, and morphine. During the study period no changes were done in surgical methods, perioperative medication routine, staffing or discharge criteria.

### Outcomes

The primary outcome of this study was the difference in PONV between the pre- and post-implementation groups as recorded in SPOR during PACU care, also referred to as postoperative phase II [16]. PONV was recorded as none, nausea, or vomiting. Secondary outcomes were differences in length of stay in the PACU, pain levels recorded on an 11-step numeric rating scale, and surgical duration as recorded in SPOR.

All participants were observed in the PACU until they fulfilled local discharge criteria. PACU nurses performed continuous clinical assessments, and documented pain and adverse events. As guidance—not an absolute requirement—patients were considered ready for discharge from PACU upon achieving a modified Aldrete score of ≥ 9 [17, 18]. In ambiguous cases, an anaesthesiologist reviewed the patient and authorised discharge. No outcomes beyond discharge from PACU were collected, and so the maximum follow-up was approximately 4 h post-surgery for almost all patients.

Selection bias was minimised by including *all* elective THA and TKA cases performed under general anaesthesia within the study years; no sampling was employed. Information bias is unlikely because all variables were retrieved from a validated national registry. Confounding was reduced through multivariable regression adjusting for type of surgery, age, body mass index (BMI), sex, smoking, American Society of Anesthesiologists (ASA) score, airway management technique, conversions from spinal anaesthesia, use of volatile agents, and duration of surgery.

### Statistical analysis

Data were analysed using version 28 of IBM SPSS Statistics and version 4.3.3 of R. Descriptive and frequency analyses were used for data description. Differences between the periods were tested with an independent sample t-test, the Wilcoxon–Mann–Whitney test, or Pearson’s chi-square test, as appropriate. Logistic and linear regression models were used for multivariable analysis. The statistical significance level was set at 95%. Pairwise deletion was applied to handle missing data.

## Results

After excluding the 1059 surgeries performed in the washout period, this study included 2316 surgeries in 2210 patients, of which 1258 were performed in the pre-implementation period (2018–2019) and 1058 were performed in the post-implementation period (2022–2023). Characteristics are presented in Table 1. ASA classification was slightly higher in the post-implementation period (*p* = 0.020), but in both periods the median value was 2 and the interquartile range (IQR) was 2–2. No bilateral procedures were performed in this study. No peripheral nerve blocks were used, however, there were 52 cases of conversion from spinal in the pre-implementation group compared to 6 in the post-implementation group (*p* < 0.001).

**Table 1:** Baseline characteristics

	Pre-implementation (*n* = 1258)	Post-implementation (*n* = 1058)	*p*-value
Type of surgery (THA/TKA)	382/876 (30.4% THA)	536/522 (50.7% THA)	< 0.001 ^a^
Male/female	556/701	465/593	0.651 ^a^
Age, years	68.9 (± 2 SD: 49.6–88.82)	69.6 (± 2 SD: 50.9–88.3)	0.08 ^b^
BMI, kg/m^2^	28.7 (± 2 SD: 19.9–37.4)	28.4 (± 2 SD: 19.6–37.3)	0.225 ^b^
Smokers	8 (0.6%)	0 (0%)	0.946 ^a^
ASA classification	2 (IQR: 2–2)	2 (IQR: 2–2)	0.025 ^c^
Airway management (intubation/supraglottic airway device)	451/807 (35.9% intubations)	457/601 (43,2% intubations)	< 0.001 ^a^
Use of volatile agents	34 (2.7%)	17 (1.6%)	0.099 ^a^
Conversion from spinal anaesthesia	52 (4.1%)	6 (0.6%)	< 0.001 ^a^

*ASA * American Society of Anesthesiologists, *BMI* body mass index, *IQR* interquartile range, *SD* standard deviation, *THA* total hip arthroplasty, *TKA* total knee arthroplasty

^a^ chi-square test, ^b^ t-test, ^c^ Wilcoxon–Mann–Whitney test

### Postoperative nausea and vomiting

Outcome data for PONV were available for 2122 cases. Some degree of PONV was reported for 89 patients (8.3%) in the pre-implementation period and 31 (3.0%) in the post-implementation period (*p* < 0.001) (Table 2).

**Table 2:** Postoperative nausea and vomiting

	Pre-implementation	Post-implementation
No nausea	973 (91.6%)	1009 (97.0%)
Nausea	88 (8.3%)	26 (2.5%)
Vomiting	1 (0.1%)	5 (0.5%)
Total cases with data	1 082	1 040

*p*< 0.001 for pre-intervention versus post-intervention (chi-square test)

**Fig. 2:**
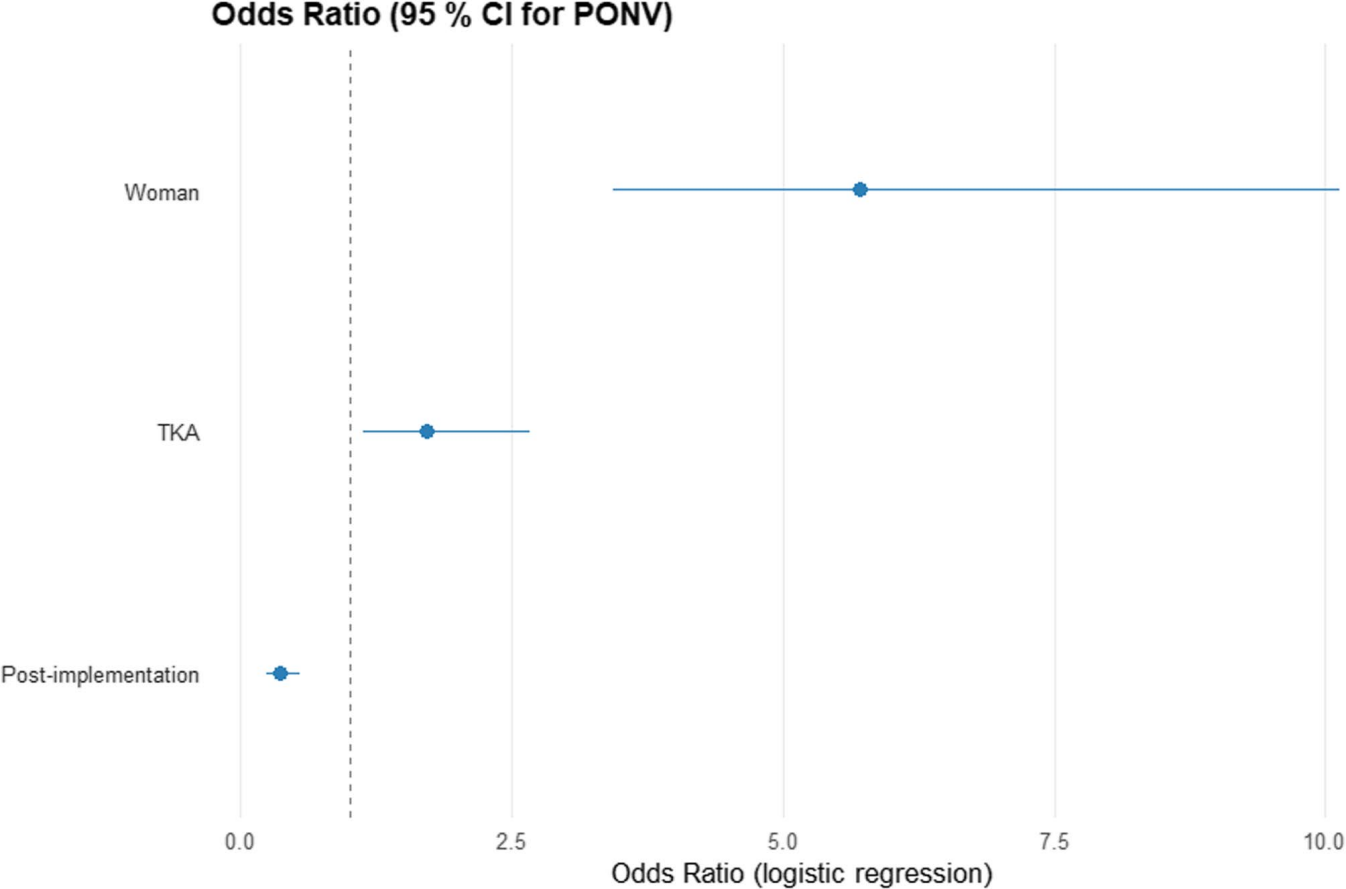
Multivariable logistic regression for postoperative nausea and vomiting (PONV)

For the multivariable regression analysis, type of surgery, age, body mass index (BMI), sex, smoking, ASA score, airway management technique, conversions from spinal anaesthesia, use of volatile agents, and duration of surgery were considered in addition to the period. However, only the type of surgery and sex were statistically significant in a univariate analysis and hence included in the final multivariable analysis. The adjusted odds ratio (OR) for PONV was 0.36 (0.23–0.55, *p* < 0.001) for the post-implementation period (Fig. 2; Table 3). No statistically significant interactions were present among the included variables. For this analysis, PONV was regarded as a binary outcome: PONV of any degree versus no PONV.

**Table 3:** Multivariable logistic regression for postoperative nausea and vomiting

	OR	95% CI	*p*-value
**Implementation period**			
Pre-implementation	*-*	*-*	
Post-implementation	0.36	0.23, 0.55	< 0.001
**Sex**			
Male	*-*	*-*	
Female	5.71	3.44, 10.1	< 0.001
**Surgery**			
THA	*-*	*-*	
TKA	1.71	1.13, 2.66	0.014

*CI *confidence interval,* OR *odds ratio,* THA *total hip arthroplasty,* TKA *total knee arthroplasty

A sensitivity analysis was performed in which all missing outcome data for PONV were set to no PONV in the pre-implementation group and to PONV in the post-implementation group. The results were still statistically significant in favour of the post-implementation group.

### Time in the post-anaesthesia care unit

Data on discharge from the PACU were available for 2203 surgeries, 1148 in the pre-implementation period and 1055 in the post-implementation period. The average time in postoperative phase II was 203 min (range: 54–1625) in the pre-implementation period and 133 min (range: 33–1546) in the post-implementation period (*p* < 0.001).

A multivariable analysis was also performed, which confirmed that the PACU time was 70 min shorter (95% CI: 61–78 min; *p* < 0.001) in the post-implementation period.

### Pain after surgery

Data on postoperative pain were available for 2057 surgeries, 1050 in the pre-implementation period and 1007 in the post-implementation period. Early postoperative pain levels ar presented in Table 4.

**Table 4:** Postoperative pain-levels

	Pre-implementation (*n* = 1050)	Post-implementation (*n* = 1007)	
NRS 1 h	6 (2–8)	5 (3–7)	< 0.001
NRS highest	7 (3–8)	5 (3–7)	< 0.001
NRS discharge	3 (2–3)	2(2–3)	< 0.001

Median (IQR), NRS = Numeric rating scale. 1 h = highest pain in the first hour, highest = highest recorded pain level during PACU-stay, discharge = pain level at dischare

### Time for surgery

The mean time for surgery was 84 min (range: 46–208) in the pre-implementation period compared to 87 min (range: 32–260) in the post-implementation period (*p* = 0.003).

## Discussion

We hypothesised that implementation of TCIe and SPV would have positive effects on PONV and duration of PACU stay. Our main finding in this study was that the post-implementation group had a statistically significantly lower frequency of PONV, and shorter time in postoperative phase II. There were statistically significant differences in pain and surgical time, but these were of unclear clinical relevance.

THA and TKA are common surgeries for osteoarthritis, and the number of patients is continuously increasing. By helping the patient to mobilise early, the length of stay can be reduced by a few days [19]. Reducing postoperative PONV and pain is important and is emphasised in Enhanced Recovery After Surgery (ERAS) guidelines, as it will benefit early mobilisation and has been shown to be beneficial for postoperative otucomes [20].

The present results indicate that in comparison to TCIp without SVP, the use of TCIe and SPV in arthroplasty produces better results in terms of PONV and length of postoperative phase II. These results are in line with some earlier findings that the use of SPV leads to a shorter time to reach Aldrete score ≥ 9, which is a criterion-based definition of the end of postoperative phase II [10]. However, other studies have not shown any effect on the early postoperative recovery [21]. To our knowledge, no comparative studies between TCIe and TCIp with these or similar outcomes have been reported.

Reducing PONV will also improve the patient’s overall experience of the hospital visit [22]. Moreover, decreasing the length of postoperative phase II also has positive effects on resource use and economics.

Longer durations of surgery have been linked to increased risk of postoperative complications [23, 24]. Our study revealed some differences in early postoperative pain and surgical time, but these differences were small and therefore unlikely to be clinically relevant, despite being statistically significant.

The present results show possible beneficial outcomes for hospitals using general anaesthesia with TCIe and SPV rather than TCIp. Not only could a change in anaesthetic method reduce costs for the hospitals in terms of lower consumption of anaesthetics and improved use of the PACU, with shorter stays meaning that more patients can be cared for, but it could also be beneficial for the patient in reducing postoperative side effects and length of stay. Implementing a new anaesthetic method might take time and resources, as hospital staff need to be trained. This study was performed in a clinical setting and included more than 1000 surgeries in the post-implementation group, showing that such an implementation can be successfully performed.

This study has both strengths and limitations. One strength is the data from SPOR, including substantial information about perioperative parameters. This made it possible to investigate distinct aspects to find differences in the groups. Another strength is the similarity between the groups in terms of gender, sex, age, BMI, and the number of surgeries included. The bispectral index was used for all patients both pre- and postimplementaion, indicating an additional benefit of SPV beyond an alternative to anaesthesia depth monitoring.

A limitation of this study is the percentage of missing data for the primary outcome in the pre-implementation group, which might have affected the outcome. There is also limited preoperative data in the registry, most problematic is the limited information on comorbidities. There was a higher proportion of TKAs in the post-implementation period, and so the type of surgery was included in the multivariable analyses. We also lacked data on actual dosage of propofol and opioids.

The overall risk of bias is low, but it cannot be ignored. While no deliberate changes in perioperative routines were implemented during the study period, potential trends—such as gradual refinement of enhanced recovery pathways or subtle adjustments in discharge practices—could theoretically have influenced the outcomes. To our knowledge, no such changes occurred at our institution during these years, yet the possibility of residual temporal confounding remains. The study period spans six years, and because SPOR does not capture all possible confounders, unmeasured factors may have influenced the results. This limitation is particularly important for the secondary outcomes, where influences beyond anaesthetic management are more likely. Moreover, the absence of intraoperative hemodynamic and pharmacological data further limits the interpretation of our findings.

Örebro University Hospital, Lindesberg is a Swedish hospital performing about 800–1 000 primary THA and TKAs yearly. The patient mix likely mirrors most Scandinavian centres, suggesting good transferability to similar settings, whereas extrapolation to centres performing fewer procedures, or to low-resource environments, warrants caution. A relatively high frequency of general anaesthesia is also noted compared to international studies [25]. The high variability in anaesthetic metods between countries, and even hospital, might limit the generalizabilty of our results.

Further research is recommended, especially regarding the effect of SPV and TCIe separately; specifically, prospective and health-economic studies. Future prospective studies should therefore include detailed pharmacological data to clarify how SPV and TCIe influence anaesthetic drug requirements and recovery profiles. Other areas of research where the techniques are implemented in different types of surgeries, as well as studies on postoperative recovery and cognitive function, would also be of interest and clinical importance. 

In conclusion, this cohort implementation study demonstrates significant benefits associated with the introduction of TCIe and SPV in patients undergoing THA and TKA. The post-implementation group experienced substantial reductions in PONV (OR: 0.36 [0.23–0.55]) and shortened duration of PACU stay. These results suggest that TCIe combined with SPV enhances early anaesthesia outcomes and may improve the likelihood of earlier postoperative hospital discharge.

## Data Availability

The datasets used and analysed during the current study are available from the corresponding author on reasonable request, considering possible legal restrictions.

## Abbreviations

ASA: American Society of Anesthesiologists
BMI: Body mass index
IQR: Interquartile range
PACU: Post-anaesthesia care unit
PONV: Postoperative nausea and vomiting
SPOR: Swedish Perioperative Registry
SPV: SmartPilot View
TCI: Target-controlled infusion
TCIe: Effect-site-targeted TCI
TCIp: Plasma-targeted TCI
THA: Total hip arthroplasty
TKA: Total knee arthroplasty
